# Emerging Sexual Transmission of *Trichophyton mentagrophytes* Genotype VII Infections, United States

**DOI:** 10.3201/eid3110.251056

**Published:** 2025-10

**Authors:** Priyanka Anand, Laura A.S. Quilter, Jessica Penney, Philip M. Polgreen, Susan E. Beekmann, Jeremy A.W. Gold

**Affiliations:** Centers for Disease Control and Prevention, Atlanta, Georgia, USA (P. Anand, L.A.S. Quilter, J. Penney, J.A.W. Gold); University of Iowa Carver College of Medicine, Iowa City, Iowa, USA (P.M. Polgreen, S.E. Beekmann)

**Keywords:** *Trichophyton mentagrophytes*, fungi, dermatomycoses, tinea, Trichophyton, sexually transmitted infections, fungal infections, Emerging Infections Network, TMVII, United States

## Abstract

*Trichophyton mentagrophytes* genotype VII (TMVII) is an emerging dermatophyte strain associated with sexual transmission among men who have sex with men. A hypothesis-generating query of US infectious diseases specialists found that 56% had heard of TMVII and 23% knew how to treat TMVII infections, underscoring a need for increased clinician education.

*Trichophyton mentagrophytes* genotype VII (TMVII) is an emerging fungus that causes dermatophytosis (particularly tinea pubogenitalis, glutealis, or faciei) and is associated with sexual transmission. Initially described in travelers returning from Thailand after sexual contact with sex workers, TMVII infections more recently were reported among men who have sex with men (MSM) in Europe (*1*–*3*). TMVII infections also have been reported in China, where cases were diagnosed in a family operating a rabbit farm, including 1 child who had Majocchi’s granuloma, a condition characterized by the formation of nodules in the skin that results from a deep fungal infection (*4*,*5*). The first US case of TMVII was reported in June 2024, after which public health authorities were alerted to several additional cases, all among MSM (*6*,*7*).

Recognizing and appropriately treating TMVII infections (typically with oral terbinafine) is essential to improve outcomes. TMVII infections might be confused with noninfectious conditions (e.g., psoriasis) and other sexually transmitted infections (*7*), and delayed treatment might result in scarring or secondary bacterial infection and continued spread (*2*). Patients with TMVII infection might seek care in sexual health clinics or HIV preexposure prophylaxis or HIV care settings, often under the supervision of infectious disease (ID) clinicians.

To help explore strategies to increase disease recognition and prompt early treatment, we polled member clinicians of the Emerging Infections Network (EIN) (https://ein.idsociety.org), a sentinel network of ID physicians and other ID specialists. This exploratory poll was not designed to represent the knowledge and practices of the membership of EIN but only to generate hypotheses for future study.

During May 2025, EIN distributed a poll that assessed clinician knowledge of TMVII to ≈3,000 member subscribers on 3 separate occasions ≈1 week apart (Tables 1,2). EIN queries are designated as non–human subjects research by the institutional review board of the University of Iowa.

**Table 1:** General awareness of TMVII infection among 117 polled member clinicians of the Emerging Infections Network, by selected characteristics, United States, 2025*

Characteristic	No. (%)
Primary setting of clinical practice	
University hospital	42 (36)
Community hospital	17 (15)
Outpatient only	15 (13)
Nonuniversity teaching hospital	10 (9)
Veterans Affairs hospital or Department of Defense	11 (9)
City, county, or public hospital	10 (9)
Children’s hospital	1 (1)
Other	11 (9)
Type of provider	
Infectious disease physician	105 (90)
Physician assistant, physician associate, or nurse practitioner	2 (2)
PharmD/Doctor of Pharmacy	5 (4)
Other	5 (4)
How did you first hear about TMVII infections?	
I had not previously heard of this issue	52 (44)
I had heard of this issue	65 (56)
Source	
CDC’s Morbidity and Mortality Weekly Report	30 (46)
Emerging Infections Network emails	27 (42)
Public health department communication	9 (14)
CDC website	8 (12)
Clinician education website	8 (12)
Clinical colleagues	8 (12)
Scientific publication	8 (12)
Other professional listserv	7 (11)
News media	6 (9)
Social media	2 (3)
Patient	1 (2)

*CDC, Centers for Disease Control and Prevention; TMVII, *Trichophyton mentagrophytes* genotype VII.

**Table 2:** Awareness of TMVII infection, diagnosis, laboratory testing, and treatment among 117 polled member clinicians of the Emerging Infections Network, United States, 2025*

Survey question	Yes	No	Not sure or not answered
In the past year, I have seen or consulted on a patient with a dermatophyte infection that was suspicious for TMVII infection	5 (4)	105 (90)	7 (6)
In the past year, I have seen or managed a patient with a laboratory-confirmed TMVII infection	1 (1)	110 (94)	6 (5)
I know when to suspect TMVII infection in clinical practice	42 (36)	42 (36)	33 (28)
If I saw a patient with potential TMVII infection, I would be able to obtain laboratory testing to determine the species	33 (28)	41 (35)	43 (37)
I know how to treat TMVII infection	27 (23)	53 (45)	37 (32)
I know how to counsel patients with TMVII infection about preventing spread	34 (29)	45 (38)	38 (33)

*Values are no. (%) respondents. TMVII, *Trichophyton mentagrophytes* genotype VII.

Of the 117 respondents, 65 (56%) reported having heard of TMVII infection. Of those, 30 (46%) reported hearing about TMVII from the Centers for Disease Control and Prevention’s Morbidity and Mortality Weekly Report (*7*) and 27 (42%) from EIN emails. Only 5 (4%) of the 117 respondents reported seeing a patient with a dermatophyte infection that was suspicious for TMVII involvement in the previous year, and only 1 respondent reported seeing a patient with confirmed TMVII infection. A total of 42 (36%) respondents stated they knew when to suspect TMVII infection, how to obtain laboratory testing specific for TMVII (33 respondents [28%]), how to treat TMVII infection (27 [23%]), or how to counsel patients about preventing spread of TMVII (34 [29%]).

The findings of this poll cannot be used to draw conclusions about the knowledge and practices of all EIN members. However, the results suggest that, although most members have heard of TMVII infection, only a minority have experience with it or know how to diagnose or treat it. TMVII is an emerging dermatophyte infection that can cause severe pain and scarring and requires prolonged treatment, particularly if diagnosis is delayed (*2*). 

Although direct microscopic examination at the point of care can help confirm a dermatophyte infection, it cannot distinguish dermatophyte species (*8*). Fungal culture can be insensitive and can require weeks before results are obtained; in addition, genotyping to identify dermatophyte species such as TMVII is not routinely available (*7*,*9*). Therefore, clinicians should be aware of TMVII and consider initiating empiric treatment with oral terbinafine (250 mg/d) in patients with consistent epidemiologic context and clinical manifestations without waiting for species confirmation (*7*).

Recognition of potential TMVII infection by clinicians can enable appropriate management and counseling, including initiation of empiric oral treatment, testing for sexually transmitted infections, and partner notification (*7*). Representative clinical images of TMVII infection are available from the Centers for Disease Control and Prevention’s Morbidity and Mortality Weekly Report (*7*). Although TMVII has not been associated with antimicrobial resistance, ID clinicians can serve an important role in recognizing and monitoring emergence of this infection in their communities. 

If the knowledge of this sample of ID physicians reflects that of the larger clinician population, then a need exists for greater education about TMVII infections and their management. Healthcare professionals who suspect a TMVII infection can contact their state or local health departments for assistance (https://www.cdc.gov/publichealthgateway/healthdirectories/index.html) and can email fungaloutbreaks@cdc.gov for assistance with recommendations and testing.
